# Surviving and Thriving in Medicine: Developing Theory‐Based Interventions for Students From Widening Access Backgrounds

**DOI:** 10.1111/tct.70076

**Published:** 2025-04-11

**Authors:** K. Gibson Smith, E. Ferguson, K. Gouveia, K. A. Walker, C. Lumsden, A. Poobalan, A. Laidlaw

**Affiliations:** ^1^ Institute for Education in Medical and Healthcare Sciences University of Aberdeen Aberdeen UK

**Keywords:** behaviour change, intervention, widening access, widening participation

## Abstract

The challenges facing students from widening participation (WP) backgrounds do not simply disappear upon entering medicine. Accordingly, it is imperative that in promoting equity in medicine, we understand how WP students may be best supported to thrive in their studies. This research aimed to develop an evidence‐based and theory‐informed intervention strategy to target student support amongst undergraduate WP students in medicine. Workshops were conducted with staff working in the medical school and students from WP backgrounds. Participants generated potential intervention ideas and critically considered the feasibility of implementation. Data analysis and intervention development were supported by the Theoretical Domains Framework (TDF) and Behaviour Change Wheel (BCW). The TDF and BCW were successfully conceptualised to structure an intervention strategy to enhance student support amongst WP students in medicine. Workshop participants identified support needs of students from WP backgrounds, and these were prioritised and used to drive intervention development. We outline two interventions that were developed from the research: adaptation of the existing personal tutor scheme and implementation of a WP peer network. The theory‐based intervention strategy outlines a foundation that could be utilised to develop and evaluate interventions to support students from WP backgrounds in medicine. This study has demonstrated how an intervention development framework (BCW) using a theoretical base can be used to develop interventions for students from WP backgrounds. Two intervention ideas were developed from the research and were designed to promote support seeking, social connection and a sense of belonging in students from WP backgrounds.

## 1 Background

Widening participation (WP) in medicine is critical to enhancing diversity within the medical community, and there has been significant investment made to promote participation in medicine amongst historically underrepresented groups. Whilst progress has been made in terms of widening access (WA) to university to study medicine amongst underrepresented groups, it has become evident that the challenges that these students face over the duration of their studies do not simply disappear once they enter medical school. For example, students from WP backgrounds have reported experiencing challenges whilst at university in relation to a lack of preparedness and struggling to fit in [1]. Accordingly, it is imperative that inclusive learning environments are embedded within medicine. These will promote equity [2] and consider how support needs may differ in accordance with individual circumstances—particularly amongst underrepresented groups. To successfully embed inclusive learning environments, we need to understand how WP students may be best supported over the course of their undergraduate medical studies.

A previous evaluation [3] of a Gateway programme, a pipeline pre‐medicine programme established in 2017, identified a key element that has been perceived to contribute to what works and enables students to successfully apply to medicine, that is, the development of supportive relationships with others. Similarly, Sartania, Aldrige and Ray [1] have stressed the importance of ensuring that students from WP backgrounds have a strong support network over the course of their studies. Whilst we know that support provision is instrumental to students from WP backgrounds in promoting flourishing, little is known about the requirements for creating a supportive environment for WP students. How may they be best supported, from their perspective, over the course of their studies and training to thrive and flourish? This research aimed to address this evidence gap and develop an intervention strategy for WP students, throughout their undergraduate academic journey.

It is critical to ensure rigour and robustness, any intervention strategy should be both evidence‐based and theory‐informed [4, 5]. The use of theory in healthcare professions education (HPE) is recognised as a means of promoting quality and rigour in research, and there are many excellent examples of how theory may be integrated into research design and interpretations [6]. Researchers studying HPE can select a plethora of theories from a variety of disciplines, including but not limited to sociology, psychology, learning and organisational approaches, to ‘frame’ and inform their research.

However, behaviour change theories are a relatively new paradigm [7] within the field of HPE research and have much to offer in terms of promoting the development of evidence based and theory‐informed innovations [8]. Therein, this research aimed to develop an evidence‐based and theory‐informed intervention strategy to support WP students' to flourish in medicine. Whilst the data presented is from one medical school, the key messages and outcomes of the study will be beneficial to those working in similar contexts where there is significant investment and innovation in WP to medicine.

*This research aimed to develop an evidence‐based and theory‐informed intervention strategy to support WP students' to flourish in medicine*.

## 2 Methodology

### 2.1 Design

This was a qualitative study with data collected from stakeholders via cocreation workshops with the aim of developing an evidence‐based and theory‐informed intervention strategy. The development of the intervention strategy was undertaken using a systematic qualitative methodology grounded in the relevant theory. The study was undertaken at a Scottish University that offers both a pre‐medicine for students from WP backgrounds (Gateway programme) and undergraduate medical programme (MBChB).

### 2.2 Theoretical Lens

Supporting WP students' to flourish in medicine requires institutional commitment [1]. Therein, it was recognised that the Capability, Opportunity, Motivation–Behaviour (COM‐B) model and the Behaviour Change Wheel (BCW) [9] underpinned by the Theoretical Domains Framework (TDF) [10, 11] were appropriate frameworks to adopt and to develop an intervention strategy. We adopted this approach as it is a unification of multiple behaviour change theories and therefore does not confine researchers to selecting just one theoretical approach.

The TDF was developed initially via a consensus exercise and is a synthesis of a plethora of behavioural theory condensed into 12 domains [10], which was later expanded to 14 [11]. The TDF is a determinant framework [10] and hence may be particularly beneficial in understanding barriers and facilitators of behaviours. The TDF has been used within HPE previously; examples include identification and exploration of factors affecting direct observation of trainees in relation to their clinical performance [12] and barriers and facilitators of effective interprofessional teamwork in operating theatres [13].

As aforementioned, the TDF can either be used as a standalone framework or be integrated within the BCW method that is a comprehensive and systematic approach to inform intervention development. TDF domains can be mapped to one of the three interrelated components specified in the BCW (see [10] for mapping). The developers outline Capability, Opportunity and Motivation; all of which, they posit, contribute to the enactment of a behaviour and therein, a system referred to as COM‐B. Components of the COM‐B can be matched to relevant intervention functions and policy categories to further inform and refine intervention development. Relevant components of the BCW can then be mapped to key Behaviour Change Techniques using the Behaviour Change Taxonomy [14].

### 2.3 Participants

All staff who taught on the MBChB at the University were invited to participate in the research. Students enrolled in the MBChB and who had completed a Gateway programme for students from WP backgrounds or who met WP criteria prior to entry (outlined in Table 1) to the MBChB were eligible to participate.

**TABLE 1:** WP criteria used with our sample.

Eligibility criteria
Applicant was resident in an area with a SIMD20 (Quintile 1) postcode as classified by the Scottish Index of Multiple Deprivation (you can check SIMD status by entering your postcode, prior to the point of entry to the MBChB, into the SIMD postcode checker)
Applicant was a young person who was care experienced (e.g., looked after by the local authority in care or care leaver)
OR—met a minimum of two from the below list:
Applicant was registered with (or eligible for) the Reach Program Scotland
First generation applicant to higher education
Applicant was eligible for the Educational Maintenance Award
Applicant attended a target school.
Applicant provided verifiable independent evidence (e.g., from School Head Teacher) of severe sustained financial hardship that is not reflected in current SIMD categorisation

*Note:* Prior to secondary school, the applicant was not schooled in English (e.g., the applicant did not speak English when starting secondary school).

We estimated that we would require 10–12 participants across a minimum of two workshops to achieve data sufficiency. This estimation was based on Malterud et al.'s [15] guidance on information power, in that we (i) required a small‐sized sample since our aim was focussed, (ii) were recruiting specific participant characteristics, for example, students who fulfilled WP criteria (as per the criteria outlined in Table below and in line with university admissions criteria), (iii) intended to utilise theory to interpret findings, (iv) expected to attain a strong dialogue with participants based on the researchers' experience of conducting qualitative research, and (v) were using a case approach to analysis.

### 2.4 Recruitment

All students and staff at the University, who taught (i.e., educationalists), on the Gateway and MBChB programmes were emailed by an administrator with details of the study—two reminder emails were sent at fortnightly intervals. Snowball sampling was also undertaken wherein interested staff and students were asked to pass on details of the study to those that might be interested using their personal contacts and networks. In addition, a medical student society disseminated details of the study via their social medial channels and internal email distribution lists. Participants provided written informed consent.

### 2.5 Data Collection

The research utilised a codesign approach [16], whereby the target population was directly involved in intervention conception (e.g., framing the problem) and design (e.g., design criteria and plan prototype). Accordingly, the codesign approach adopted in this research was conducted in multiple stages with equal input from stakeholders including WP students enrolled on the MBChB and educationalists. Two student interns were involved in the design of the recruitment strategy and workshops in addition to assisting with workshop delivery, data collection and analysis. Two workshops were undertaken with WA students and educationalists involved in the delivery of the MBChB. Each workshop was audio recorded using a digital audio recorder.

The workshops endeavoured to determine key support areas within the MBChB that currently work along with identifying areas that require improvement to formulate ideas on what might work to support WP students throughout their studies going forward. Workshop participants were asked to develop fictional characters based on their experiences (as either a student from a WP background or as a staff member in their professional roles). These fictional characters were based on the idea of ‘personas’ as has been widely adopted in user design research and perceived to be beneficial in focussing efforts to develop solutions on the needs of the end user [17]. This particular approach was adopted, as it permitted workshop participants to consider the individual needs of the proposed user, for example, in this case, a student from a WP background. We also felt adoption of this method safeguarded student's experiences—the use of the persona allowed them to draw on their experiences without specifically disclosing their own background [18].

*The workshops endeavoured to determine key support areas within the MBChB that currently work along with identifying areas that require improvement to formulate ideas on what might work to support WP students throughout their studies going forward*.

Using the fictional character, participants were encouraged to think about specific challenges the character may face in relation to their background. The definition of WP is broad and hence, focussing on a specific character and the challenges they faced allowed participants to narrow their focus. This was critical as workshops were time limited due to funding resource and research staff time. The workshops led to the identification of key priority areas and associated target behaviours, with which to focus an intervention strategy, the generation of potential intervention ideas, and consideration to the feasibility of implementation. The intervention ideas were developed separately by staff and student groups in siloed workshops. This was purposively done to avoid power imbalance and acquiescence.

### 2.6 Data Analysis

Relevant excerpts were identified, extracted, and transcribed from the workshop audio recording by two researchers (K.G.S. the PI, who is a chartered psychologist with expertise in behaviour change, and a research assistant, M.D., who has a background in psychology) and coded in accordance with the 14 domains outlined in the TDF, for example, quotes matched deductively to each domain of the TDF. The aim of the coding exercise was to identify key barriers and facilitators that promote support for WP students in their medical studies in relation to each of the target behaviours identified by workshop participants as being of significant importance. TDF domain data were then mapped to components of the BCW, in accordance with each stage of the wheel, by K.G.S., and independently checked by A.L., who also has expertise in behaviour change. Disagreements in coding were resolved via discussion. The mapping exercise was then used to formulate the intervention strategy representing each of the target behaviours identified as priority areas in the workshops [18].

### 2.7 Ethical Review

Ethical approval was granted by the School of Medicine, Medical Sciences and Nutrition Ethics Review Board (SERB/2023/1/2469) in February 2023.

## 3 Results

**FIGURE 1:**
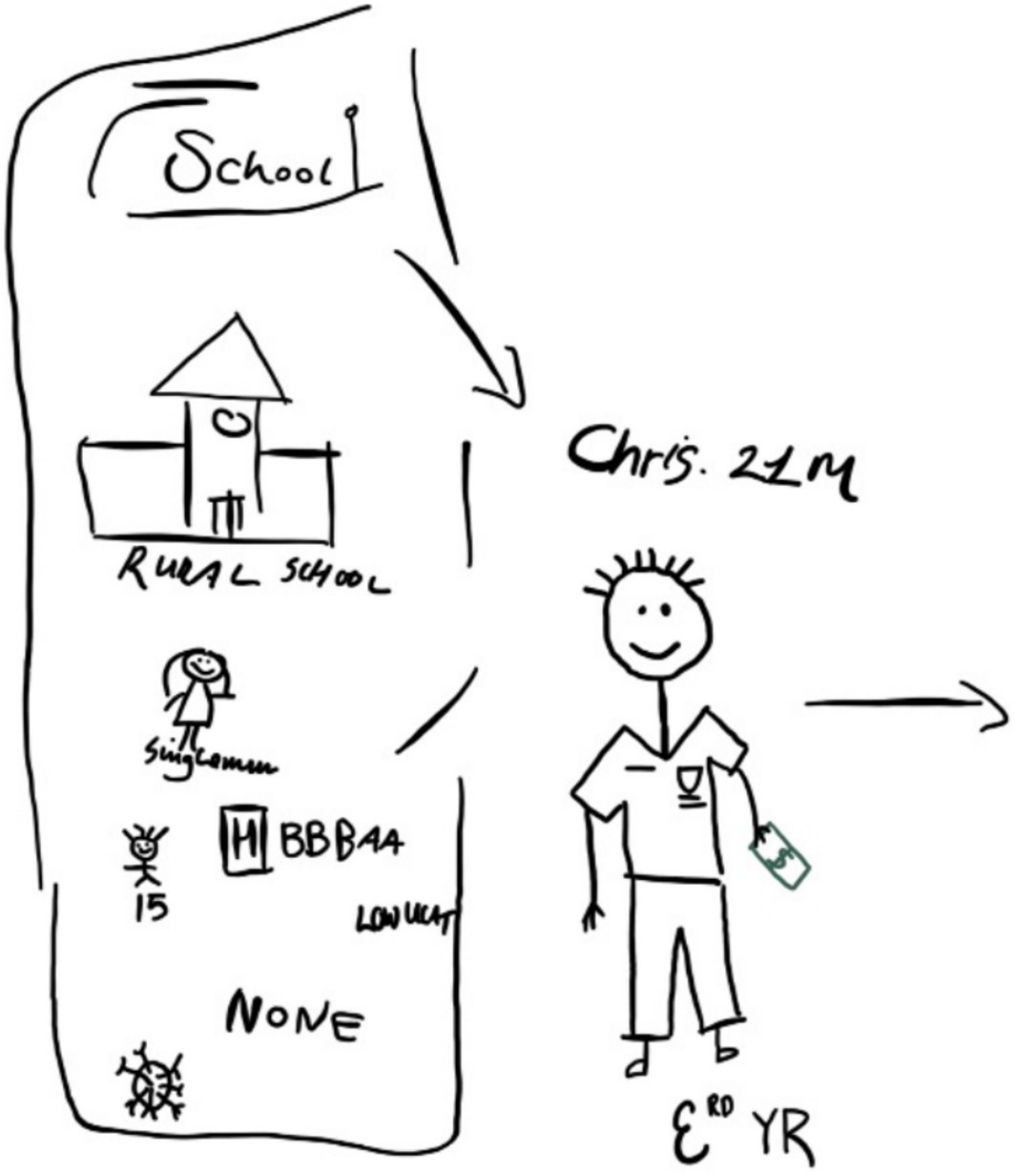
Fictional character developed in workshop.

Four groups (two staff and two student groups) developed characters (e.g., Figure 1) and intervention ideas across three different workshops. Intervention ideas were generated out of the priorities identified by workshop participants in relation to the challenges facing the fictional characters they developed. Participants identified a key target behaviour that is related to their priority area, and interventions were developed around promoting/changing these. We outline details of two of the intervention ideas and the behaviour they address below.

*Intervention ideas were generated out of the priorities identified by workshop participants in relation to the challenges facing the fictional characters they developed*.

### 3.1 Intervention 1: Adapting Personal Tutor Scheme to Better Support Students From WP Backgrounds

The intervention idea developed from the staff workshop specifically targeted: *developing positive supportive relationships with staff*. Relevant TDF domains were coded as follows: knowledge (e.g., of the local context); skills (e.g., developing study skills); environmental context and resources (e.g., not being able to visit family regularly); social influences (e.g., not being familiar with university culture); professional/social role and identity (e.g., not having family connections in medicine); beliefs about capabilities (e.g., not having confidence in ability to do medicine); beliefs about consequences (e.g., stigma associated with seeking student support); reinforcement (e.g., reinforcing that it is okay to seek help) and emotion (e.g., stress from working too much). As identified in the TDF analysis, participants discussed that students often struggled with the stigma of seeking support at university.

Participants considered one way to overcome this was via the pre‐existing personal tutor scheme. The group outlined that it was critical for students to develop trusting and open relationships with personal tutors. Although it was suggested that it might be ideal to match students from WP backgrounds with staff members who had similar experiences, they realised that this would be difficult to implement due partly to student numbers and perhaps because personal tutors may have not declared their backgrounds. Therefore, they suggested a mechanism was developed whereby personal tutors could share good practice and experiences about challenges that students from WP backgrounds may face. It was proposed that a means of doing this would be via revising the existing handbook for personal tutors and also, setting up contracts with students to agree expectations of the relationship.

Participants also felt that it was important that personal tutors were aware if any of their students fulfilled WP criteria upon entry—information that they thought could be passed onto them by administrative staff. Lastly, they felt that the current expectations around personal tutors meeting students on a once per semester basis were too infrequent for students facing challenges and perhaps this could be tailored in accordance with need to ensure students were adequately supported.

A specific intervention outlining how the development of positive relationships with staff could be fostered is presented below (Table 2) and encompassed the aforementioned points. Key components of the intervention would be to increase knowledge amongst personal tutors of challenges facing students from WP backgrounds; to signpost students to relevant student support services and resources within the local community; to frame support seeking as positive; and to provide guidance on how to seek support when needed.

**TABLE 2:** Intervention 1: adapting existing personal tutor scheme to better support students from WP backgrounds.

Intervention functions	COM‐B components served by intervention functions	Behaviour Change Techniques to deliver intervention functions	Policy categories through which Behaviour Change Techniques can be delivered	Intervention strategy
Education; Persuasion; Environmental Restructuring; Training; Modelling.	Psychological capability; Physical opportunity; Social opportunity; Reflective motivation; Automatic motivation	• **Prompts/cues**: signposting to student support services; • **Credible source**: increasing knowledge of personal tutors on WP •**Framing/reframing**: framing support seeking as a positive step in promoting wellbeing and developing coping strategies to benefit long run **Demonstration of the behaviour**: advising on how to seek support	Service provision Guidelines	Adapting current personal tutor scheme to match students from WP backgrounds to personal tutors with experience and/or expertise in WP. Sharing of good practice amongst personal tutors in order to increase pool of personal tutors. Promoting more frequent contact with students and advising personal tutors of students who fulfil WP criteria upon entry.

### 3.2 Intervention 2: Developing a WP Peer Support Network

This intervention specifically targeted: *Promoting a sense of belonging in students from WP backgrounds*. TDF analysis identified the following domains: skills (e.g., learning to use different study techniques suitable to medicine); environmental context and resources (e.g., experience of culture shock); social influences (e.g., peer comparison to those with financial security—not feeling that you can do as well as others); professional/social role and identity (e.g., issue with developing relationship with mentor from more privileged background) and emotion (e.g., feeling isolated from others). Students discussed that they felt it was important to have others who were more senior to them and who had a WP background to act as role models and mentors to students in earlier years.

Participants highlighted how pursuing career aspirations as a student from a WP background looked different. They advised that costs were often a significant barrier, for example, in relation to intercalation (funding an extra year of living costs) or gaining research experience (which is often paid less than their part time job, hence making it unaffordable for them to undertake). Participants were conscious of the negative impact this could have on their competitiveness when it came to specialty choice and training. In addition, they discussed how they were concerned about getting into specialty training in terms of the ‘how’ of it, and also, what the next steps were for them. They felt that they are now seeing role models in medicine, with similar backgrounds to themselves, and connecting with them was key to promoting a sense of belonging.

Participants proposed a university WP to medicine peer support group should be established whereby MBChB students in their early years were matched with a senior medical student or trainee with a similar background. It was suggested that the network would provide support for students from WP backgrounds via regular events and informal meetings with those more senior.

A specific intervention outlining how a sense of belonging could be promoted amongst students from WP backgrounds is presented below (Table 3) and encompassed the aforementioned points. Key components of the intervention would be to establish a WP to medicine peer support network for MBChB students; prompt support seeking where necessary in students and provide students with role models who had similar backgrounds to them who were at a more senior stage of their studies/training.

**TABLE 3:** Intervention 2: Implementation of a WP peer support network.

Intervention functions	COM‐B components served by intervention functions	Behaviour Change Techniques to deliver intervention functions	Policy categories through which Behaviour Change Techniques can be delivered	Intervention strategy
Education; Training; Environmental Restructuring; Modelling.	Psychological capability; Physical opportunity; Social opportunity; Reflective motivation; Automatic motivation	**Prompts/cues**—emails to prompt joining support network; prompts from senior members to seek necessary supports **Instruction on how to perform a behaviour**—instructing how to seek support **Restructuring the physical environment**—implementation of a WP support network **Demonstration of the behaviour**—role modelling	Communication Service provision Guidelines Environmental/social planning	Development and implementation of a WP support network for students from WP backgrounds. The network will provide support for students from WP backgrounds via regular events and informal meetings with senior medics and trainees from WP backgrounds

## 4 Discussion

To the best of our knowledge, this is the first study to use an intervention development framework (Behaviour Change Wheel) to develop an intervention strategy to support the retention, and thriving, of students from WP backgrounds. The strategy outlined two separate intervention ideas to promote: the development of supportive relationships with academic personal tutors via adaptation of an existing scheme, and a sense of belonging amongst students from WP backgrounds via implementation of a WP peer support network.

*The strategy outlined two separate intervention ideas to promote: the development of supportive relationships with academic personal tutors via adaptation of an existing scheme, and a sense of belonging amongst students from WP backgrounds via implementation of a WP peer support network*.

Our findings demonstrate how relevant intervention development frameworks may be conceptualised by healthcare professions educational researchers to support the development of educational interventions for students in higher education settings and crucially, highlights the types of interventions which are perceived by students from WP backgrounds to be beneficial in their medical education journey and which could be implemented in similar contextual settings where WP is a significant focus. Whilst the types of interventions proposed are not necessarily new or novel, they were perceived to be significant to students from WP backgrounds in supporting them over the course of their studies. They highlight how current provisions in undergraduate medical education do not necessarily serve the needs of students from diverse backgrounds and may require adaptation to promote equity. They also speak to the systemic social injustices that are embedded in medical education today and that may detract from the creation of inclusive learning environments.

The interventions developed in this study focussed on developing supportive relationships with academic staff, in the form of personal tutors, and with students and senior clinicians from similar backgrounds. The focus of these interventions on support provision resonates with previous research [3] in WP to medicine, where the importance of developing supportive relationships with academic staff and peers was highlighted as a key mechanism in supporting positive transitions in medicine. The need for students from WP backgrounds to feel ‘connected’ with those from similar backgrounds when in medical school has been previously [1] established and hence, the focus of the suggested interventions in this study on fostering social connections with others who share similar trajectories into medicine is perhaps unsurprising.

It is well established that social inequality is reproduced in higher education settings [19], and our findings on the need to connect with those with similar backgrounds suggest that this is perhaps reflected to some degree in the student psyche. We could consider this as a direct reflection of the higher education mainstream culture in which these students find themselves embedded in where they, out of necessity, feel they need to cluster in groups with those that they share a similar background (homophily). Indeed, Mavor et al. [20] suggest that developing a strong sense of shared identity with other medical students may buffer against the effects of stress. However, conversely, Lin [21] has suggested that group clustering around shared characteristics could potentially lead to the formation of resource poor social networks that lack the same social capital as groupings that form heterogeneously, which by contrast, tend to be resource rich. Hence, it is imperative to strike a balance on fostering social connectedness amongst students from WP backgrounds whilst also promoting upwards social mobility in terms of fostering connections in medicine which allow them to pursue their career ambitions.

Students and staff in our workshops stressed that a key component of the interventions they developed was to ensure that students were encouraged, and directed, to relevant support. The challenges facing some students from WP backgrounds do not simply disappear upon entry to university. For example, some students, perhaps who are estranged from family or care‐leavers, do not have access to the same family support networks as others. To exemplify, research [22] into the experiences of care experienced students in higher education has highlighted the importance of having a supportive adult within the institution who had knowledge of that young persons' background and provided ongoing support which was relational and authentic.

Accordingly, whilst the students in this study recommended implementation of a WP peer support network to facilitate enhancing their social network and connectedness, caution should be exercised that the separation of students from the traditional entry student body does not sacrifice their inclusion in wider, beneficial social networks that promote social advancement [19]. However, with that said, due regard should also be given to the ongoing challenges that some students face over the course of their educational journey and their related ongoing support needs.

The aforementioned intervention ideas speak to the importance of promoting a sense of belonging amongst students from underrepresented backgrounds within medicine. Indeed, ‘belonging’ is a concept gaining increasing prominence within the literature [23, 24]. However, as has been cautioned [24]: supporting students to ‘fit in’ to dominant social and cultural norms of institutions ‘… can perpetuate the dominant social discourses, with the potential of framing traditionally underrepresented students as underprepared for the rigours of medical school’. We were cognisant of this when developing the study initially and hope, that by undertaking a codesign approach to intervention design, we have moved beyond the discourse of encouraging students to ‘fit in’ to the dominant discourse to promote a sense of ‘belonging’. We have made efforts, by involving students in research design, delivery and interpretation, to harmonise institutional and student discourses to ensure student support is more equitable to those from WP backgrounds.

Whilst the intervention development framework, BCW [9], has allowed us to develop an intervention strategy to support students from WP backgrounds in their journey through medicine, the intervention ideas developed would benefit from further refinement with both staff and students to ensure that they promote social justice and equity. In addition, they may require further tailoring to ensure that they are institutionally feasible, for example, with increasing student numbers and online learning. Once refined and implemented, they will need to be evaluated. Evaluation should be supported by a relevant theory such as the realist approach that has previously been used to evaluate interventions in WP (e.g., [3, 25].

## 5 Conclusion

This study has demonstrated how an intervention development framework (BCW) can be used to develop interventions for students from WP backgrounds. Two intervention ideas were developed from the research with staff and students and were designed to promote support seeking, social connection and a sense of belonging in students from WP backgrounds. Whilst we support further development and implementation of the interventions, we would caution that higher education institutions need to ensure balance in terms of implementing student support interventions and creating an inclusive climate. Accordingly, consideration should be given to the implementation of interventions that are both attuned to the needs of the students they are targeting whilst also being mindful that they do not, in their very essence, perpetuate existing social inequalities.

## Author Contributions

**K. Gibson Smith:** conceptualization, investigation, funding acquisition, writing – original draft, writing – review and editing, methodology, validation, visualization, project administration, formal analysis, data curation, supervision, resources. **E. Ferguson:** methodology, data curation, writing – review and editing, project administration. **K. Gouveia:** methodology; data curation; project administration, writing – review and editing. **K. A. Walker:** conceptualization, funding acquisition, writing – original draft, writing – review and editing, methodology. **C. Lumsden:** conceptualization, funding acquisition, writing – original draft, writing – review and editing. **A. Poobalan**: methodology, conceptualization, writing – review and editing, funding acquisition. **A. Laidlaw:** conceptualization, investigation, funding acquisition, writing – original draft, writing – review and editing, methodology, validation, formal analysis.

## Ethics Statement

Ethical approval was granted by the School of Medicine, Medical Sciences and Nutrition Ethics Review Board (SERB/2023/1/2469) in February 2023.

## Conflicts of Interest

The authors declare no conflicts of interest.

## Data Availability

Research data are not shared.

## Appendix A

**Table:** Table A1 WP criteria as per student admissions at University of [anonymised].

**Eligibility criteria**
Applicant was resident in an area with a SIMD20 (Quintile 1) postcode as classified by the Scottish Index of Multiple Deprivation
Applicant was a young person who is care experienced (e.g., looked after by the local authority in care or care leaver)
**OR—met a minimum of two from the below list**
Applicant was registered with (or eligible for) the Reach Program Scotland
First generation applicant to higher education
Applicant was eligible for the Educational Maintenance Award
Applicant attended a local target school as identified by the university as having low progression rates to Higher Education
Applicant provided verifiable independent evidence (e.g., from School Head Teacher) of severe sustained financial hardship that was not reflected in SIMD categorisation

*Note:* Prior to secondary school, the applicant was not schooled in English (e.g., the applicant did not speak English when starting secondary school).
